# P-966. Infectious Disease Enrichment Program: Medical Student Accuracy in Pathogen Identification

**DOI:** 10.1093/ofid/ofae631.1156

**Published:** 2025-01-29

**Authors:** Lauren Bernard, Vincent T Brown, Thelma M Achufusi, Nhu Le, Juliana Sherchan, Astrid Widjaja, Richa Beher, Devang M Patel

**Affiliations:** University of Maryland School of Medicine, Baltimore, Maryland; University of Maryland School of Medicine, Baltimore, Maryland; University of Maryland School of Medicine, Baltimore, Maryland; University of Maryland School of Medicine, Baltimore, Maryland; University of Maryland School of Medicine, Baltimore, Maryland; University of Maryland School of Medicine, Baltimore, Maryland; University of Maryland School of Medicine, Baltimore, Maryland; University of Maryland School of Medicine, Baltimore, Maryland

## Abstract

**Background:**

There has been increased interest in identifying learning strategies that promote long-term retention of medical school content. In 2022, an extracurricular clinical microbiology project called “Pokébug'' was created by the Infectious Disease Interest Group (IDIG) to encourage such retention among preclinical students at the University of Maryland School of Medicine (UMSOM). Students are given “pseudo-case presentations” including five clues such as pathogen route of entry, clinical symptoms, and treatment. At the end of the year, the student with the highest total correct points in each class receives a gift card.

**Table 1.**
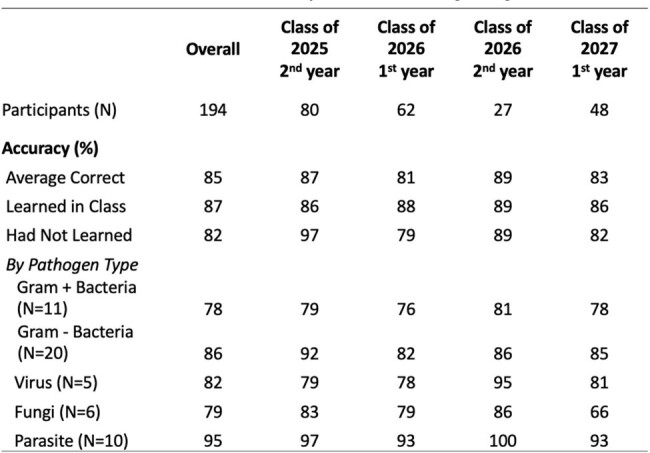
Medical Student Accuracy in the Pokébug Program

**Methods:**

Here, we studied students’ accuracy as a measure of their long-term retention of clinical microbiology material. Primary aims were to study accuracy: a) overall b) across different pathogen types c) among pathogens that had been learned in class versus not d) temporal variation.

**Results:**

A total of 194 medical students from 3 classes participated in at least one of the 52 weekly quizzes. Students demonstrated marginally higher accuracy among pathogens that had been previously taught in class (87%) versus those they had not (82%). The highest accuracy was observed for parasitic pathogens (95%), while the lowest accuracy was observed for gram positive bacteria (78%). Student accuracy improves over time, which may be due to the project’s incentive structure.

**Conclusion:**

These findings support the conclusion that Pokébug reflects long-term retention of course material and can guide curricula improvements (e.g., additional time could be given to gram-positive bacteria, employ similar active learning strategies in other content areas to strengthen students’ long-term retention). Additionally, IDIG may want to reevaluate the incentive structure to maximize longitudinal student participation.

**Disclosures:**

**All Authors**: No reported disclosures

